# African swine fever risk and plant‐based feed ingredients: Canada's approach to risk management of imported feed products

**DOI:** 10.1111/tbed.14281

**Published:** 2021-09-12

**Authors:** Sharon Calvin, Amy Snow, Egan Brockhoff

**Affiliations:** ^1^ Animal Health Risk Assessment and Intelligence Section Canadian Food Inspection Agency Ottawa Canada; ^2^ Foreign Animal Disease Section Canadian Food Inspection Agency Ottawa Canada; ^3^ Canadian Pork Council Ottawa Canada

**Keywords:** African swine fever, biosecurity, feed, import, risk analysis, risk mitigation

## Abstract

As a result of unprecedented spread of African swine fever (ASF) since 2018, Canada has taken additional steps to prevent introduction of the virus. While the role of plant‐based feed in the transmission of ASF is not completely understood, it was identified that no mitigation measures were in place to address this uncertain risk. A risk analysis process was conducted with collaboration between government and industry, including an assessment of the costs and benefits of various risk mitigation options. Using existing legislative tools, requirements must now be met before the importation of plant‐based feed ingredients of concern is permitted. Even with an uncertain risk, countries such as Canada that would suffer severe consequences should ASF be introduced, need to consider appropriate, risk‐based mitigation measures.

## 1 INTRODUCTION

African swine fever (ASF) is a contagious viral disease that only affects pigs and for which there is no treatment or vaccine. ASF has a high mortality rate and continues to spread globally at an alarming pace. Originating in Africa in the 1900s, it has now spread to many countries in Europe and Asia (Blome et al., 2020; Costard et al., 2013). ASF has never been reported in Canada but as the global viral load increases, risk of introduction to Canada goes up.

While ASF does not cause disease in humans and is not a food safety risk, its impacts on trade are significant. One positive case in Canada would stop all hog and pork product exports immediately, with markets taking months to years to reopen. Given that Canada exports 70% of its hog production (including live pigs, germplasm and pork/pork products), this interruption in trade would be a devastating blow to a 24 billion CAD industry.

In 2018, the emergence of ASF in Southeast Asia and extraordinary international spread heightened concern about the potential risk to Canada's pork industry, should ASF be introduced to the country. This concern has resulted in unprecedented prevention and preparedness activities being collaboratively undertaken within Canada, with the federal government, provincial/territorial governments and industry participating. The overarching objective of the collective efforts is to prevent ASF from being introduced to Canada and if it is, to be as prepared as possible to minimize the impact of this devastating disease.

One of the early activities that was conducted was the assessment of the possible pathways of ASF entry into Canada, including the identification of existing mitigating measures and gaps. Similar assessments of introduction pathways have been conducted by others (Beltran‐Alcrudo et al., 2019; DEFRA, 2018; Guinat et al., 2016; USDA, 2019). Relevant to this discussion, the potential for feed ingredients not of animal origin, and/or their associated packaging, to introduce foreign animal disease viruses of concern was raised due to new evidence from recent research and the ongoing international spread of ASF (Niederwerder, 2021). Although details pertaining to the role of feed in disease transmission were still lacking, and this pathway of entry was considered to be a lower risk than other pathways, it was recognized that no risk mitigation measures were currently in place. Therefore, there was collective interest in further exploring this risk to Canada, given the export dependency of the Canadian pork industry and the severe consequences that an ASF introduction would have on the Canadian economy. It was concluded that further analysis on this pathway would inform risk decisions related to disease prevention.

The World Organization for Animal Health (OIE) describes how to conduct risk analysis relating to animal health import and divides the process into four components: hazard identification, risk assessment, and risk management, with risk communication occurring throughout (OIE, 2019). This was the approach applied by the Canadian Food Inspection Agency (CFIA) in further evaluating non‐animal‐origin feed as a risk. Notably, the OIE mentions the importance of assessing risk associated with various products (including feedstuffs).

## 2 RISK ANALYSIS PROCESS

### 2.1 Hazard identification and risk assessment

The hazard identified in this case was ASF virus associated with the importation of feed ingredients not of animal origin and/or their associated packaging from countries of concern. Contaminated plant‐based feed and feed ingredients have been implicated in the spread of porcine epidemic diarrhoea virus (PED), and epidemiological associations between ASF outbreaks and contaminated plant‐based feed have been made in Romania, Latvia and Estonia (Niederwerder, 2021). Further refinement was conducted as part of the risk assessment and management step. It was determined that plant‐based feeds posed the greater risk, and that synthetic products, such as vitamins and minerals, posed a comparatively lower risk.

**FIGURE 1:**
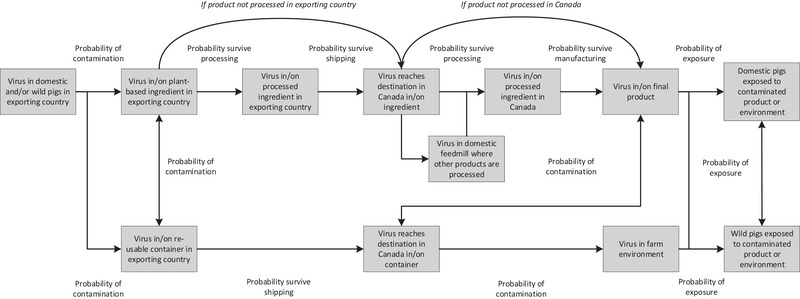
Scenario tree for introduction of African swine fever virus into Canadian swine via imported feed ingredients not of animal origin

A scenario tree was developed to describe the introduction of ASF into the Canadian domestic or wild pig population via this pathway (Figure 1). For feed ingredients to be considered a hazard, a number of steps are required. First, virus excreted by infected animals must contaminate feed ingredients or ingredient containers in the country of origin. Containers may contaminate ingredients and vice versa. If the product undergoes processing in the country of origin, the virus must survive processing or contaminate the ingredient post‐processing.

The ingredient is then shipped to its destination in Canada, and the virus must survive the environmental conditions for the length of the shipping process. Virus must then arrive in Canada in or on an ingredient or container. Some ingredients may then undergo processing in Canada, as well as manufacturing into a final feed product, and the virus must survive these steps. Alternatively, the virus contaminates the feed mill or other facility, where it then contaminates other feeds, such as those of Canadian origin. Final feed products in Canada may contaminate containers and vice versa.

Contaminated feed products or containers must then expose domestic pigs in Canada to the virus. This may occur directly if products are used as a swine feed, or indirectly via wild pigs who may become exposed by gaining access to feed used in an outdoor environment. Even if the feed product is not contaminated, virus could reach the farm environment in or on a contaminated container.

There are many potential points of contamination of ingredients in the country of origin (before, during and after processing). This can include the use of manure as fertilizer, contaminated irrigation sources, the movement of people or fomites between farms, contamination by wild pigs, and cross‐contamination during processing, storage and transport. It was determined that the information required to analyse this probability is difficult to obtain without an on‐site visit.

The assessment concluded that many aspects of product processing may affect the probability that viable virus survives, including the use of washing, solvents, changes in pH, drying and heating. Rigorous thermal processing is considered one of the best methods to inactivate foreign animal disease viruses. However, the effect of heat is dependent on the medium, and very little research was found specifically on feed. Initial research suggests that high temperatures (>80°C) can inactivate the virus in feed in just a few minutes (Selyaninov et al., 2014). ASF is also inactivated by heat treatment at a temperature of 70°C for 30 min in meat, or 60°C for 30 min in serum and body fluids (Center for Food Security and Public Health, 2019). However, the effect of more moderate thermal processes (i.e., less than 60°C) is less clear. With respect to pH, it is unlikely that any processes reach a pH sufficiently low to inactivate ASF, since extremely low pH would render feed unpalatable and unsafe.

Research has shown that ASF can maintain infectivity in various feed ingredients during the length (30 days) and environmental conditions simulating a Trans‐Atlantic trip from Europe to the United States (Dee et al., 2018). For a full assessment, further research is required on the survival of ASF in the ingredients of concern and under the environmental conditions and timing of a journey from affected countries to Canada. Since the virus survived on most ingredients tested, and in the stock virus control, it is assumed at this point in time that the virus will survive shipping equally in all ingredients imported.

There was a high level of uncertainty associated with many of the probabilities in this pathway, especially those related to contamination of ingredients in source countries, making it difficult to determine the actual probability of introduction. However, future updates in the available scientific evidence will result in reconsideration of the risk.

Despite the uncertainties, it was concluded that plant‐based feed ingredients produced in rural areas are more likely to be exposed to ASF than synthetically produced feed ingredients. For synthetic products, the lower risk is due to a lower likelihood of source contamination, extensive processing involving fermentation and/or high temperatures, greater oversight resulting in lower likelihood of cross‐contamination, and lower inclusion rates in diets (EFSA, 2011; Jones et al., 2018; SHIC, 2019). Synthetic products are more likely produced in facilities within industrial areas and facilities manufacturing synthetically produced feed ingredients are also more likely to be owned by large multi‐national companies that export widely and, as such, be subject to the oversight of various countries. This assumes that companies that produce vitamins and minerals for feed are not producing porcine blood products in the same facilities (e.g., blood meal, plasma, serum), which would present a risk for cross‐contamination.

As part of the assessment, 5 years of import data on plant‐based feed ingredients from countries affected by ASF were reviewed and characterized. This included an analysis of quantity of ingredient imported, processing in source countries and/or Canada, type of packaging, and end use. Most of the imported product was identified as organic. Importing cereal grains from overseas is expensive, and likely only makes sense for niche markets. The volumes imported per year were determined to be small, and only a small number of importers acquire these products (see Table 1). It was considered that importers will be drawn to whichever global sources are least expensive, and this is constantly changing.

**TABLE 1:** Mean (and range) volume, number of shipments, and number of importers of plant‐based feed ingredients into Canada over five fiscal years (2014/15 to 2018/19) from ASF‐affected countries

Ingredient imported	From ASF‐affected countries Yearly mean (range)		
	Volume (metric tons)	# Shipments	# Importers
Corn	17,833 (7284–27,160)	69 (40–112)	6 (3–8)
Whole soybean	12,348 (428–34,675)	40 (9–84)	4 (3–5)
Soybean meal	450 (40–1376)	9 (1–25)	2 (1–3)
Canola meal	599 (0–1422)	4 (0–10)	1 (0–1)
Flax seed meal	342 (151–549)	6 (2–10)	2 (1–3)
Sunflower meal	74 (0–309)	1 (0–3)	1 (0–2)
Corn gluten meal	20 (0–80)	1 (0–3)	0 (0–1)
Wheat	2246 (0–6545)	16 (0–57)	1 (0–2)
Barley	179 (0–632)	1 (0–3)	0 (0–1)

*Note*: Table 1 contains import data up to November 2018. Since that time, new countries have reported ASF but import data from those countries are not reflected in the table. However, any countries reporting ASF are subject to the import requirements.

A review of available information, as well as information obtained from industry engagement, indicated that the conventional processing of oilseed meals likely involves sufficient heat to inactivate ASF and this also applies to most organic processing (i.e., mechanical extraction). For example, cooking typically occurs at temperatures of 60–75°C, followed by oil extraction at temperatures of approximately 100°C or more for at least 10 min (Canadian Oilseed Processors Association and Canadian Food Inspection Agency, 2015). This processing may occur in source countries or after importation to Canada. The normal processing of corn, however, does not include these high temperatures, and neither does the processing associated with cold‐pressed oilseeds, unless the product is pelleted or extruded prior to being fed to livestock.

Cross‐contamination pre‐ and post‐processing is also a potential risk if good manufacturing practices are not followed, as is the practice of adding hulls, screenings or other materials to the product after thermal processing. It was determined that most of these ingredients are shipped in bulk packaging, adding to the potential for cross‐contamination.

Many grains and oilseeds, and associated meals, are regularly used in pig rations at moderate to high inclusion rates. Therefore, the level of exposure of an individual pig to a contaminated imported batch could be significant.

The following risk factors were identified for the potential spread of ASF with plant‐based ingredients:

- Proximity to infected zones and use of manure as fertilizer
- Minimal processing (e.g., corn) or processing that does not involve high temperature heat treatment (e.g., cold‐pressed oilseed meals)
- The use of untreated hulls or screenings as livestock feed, either on their own, added back to oilseed meals after heat treatment steps, or as an ingredient in the production of other feeds
- Reusing bulk containers without appropriate cleaning

### 2.2 Risk management

#### 2.2.1 Considerations

Canada already had strong import controls in place that address the highest risk pathways for ASF introduction, such as importation of live animals, animal products/by‐products, and feed containing animal ingredients; however, pre‐existing import measures were not in place for plant‐based feed ingredients. In addition, through various mechanisms (including strategic use of detector dog capacity and aggressive traveller awareness campaigns), attention has also been paid to preventing the illegal importation of products that are undeclared at the border. While potential risks posed by plant‐based feed ingredients are not as high as other aforementioned known risk pathways, potential introduction via this route was raised as a concern. In making risk decisions, the likelihood of agent entry into Canada, exposure of susceptible populations in Canada, and consequences of introduction were all evaluated. Given the serious consequences that an ASF introduction would have in Canada, the CFIA undertook action to mitigate the risk. The CFIA worked collaboratively with other government departments and industries to address the potential gap in risk mitigation.

Based on data that was collected, the volume of imported plant‐based feed ingredients and the number of associated importers was identified to be relatively small, and the focus was on importation to serve the organic market.

In addition to relatively small volumes and number of importers, plant‐based feed ingredients sourced directly from countries affected by ASF were identified to be predominantly imported through a small number of marine ports.

The OIE indicates that 'the objective [of risk management] is to manage risk appropriately to ensure that a balance is achieved between a country's desire to minimise the likelihood or frequency of disease incursions and their consequences and its desire to import commodities and fulfil its obligations under international trade agreements' (OIE, 2019). Following this principle, it was concluded that the application of new import requirements needed to take into consideration an evaluation of the various costs and benefits to multiple stakeholders, including costs associated with a potential outbreak of ASF and the costs associated with preventive control measures. Decisions needed to be made to select measures that had the appropriate impact in effectively reducing the level of risk in the most cost‐effective manner possible.

#### 2.2.2 Risk management decision

It was identified that the current gap in risk mitigation required a staged, multi‐prong approach and it was also recognized that these measures strengthened Canada's overall level of protection against ASF but were not intended to reduce the level of risk to zero.

In the very short term, CFIA used targeted communication with importers to facilitate compliance promotion.

In the short to medium term, the CFIA used existing legislative authorities (the ability to declare secondary control zones to reduce the threat of disease introduction) to necessitate import requirements for select plant‐based feed ingredients originating from countries of concern for ASF entering Canada through one of the six marine ports of entry that were identified. This approach can be adjusted as further information on the level of risk posed by these products emerges.

A risk ranking of potential products was completed and the plant‐based feed ingredients to be controlled included unprocessed grains, oilseeds and their associated meals, imported with the intent for use in livestock feed. These commodities were selected because, in the case of the unprocessed products, they undergo little or no processing or treatment prior to export to Canada, which leads to a greater likelihood of potential disease introduction. In the case of meals, the practice of cold‐pressing or adding untreated material back in after processing also leads to a greater likelihood of potential disease introduction. The standard processing practices of the other commonly imported non‐animal‐origin feed ingredients (such as vitamins and minerals) were determined to be acceptable risk mitigation. It was determined that the new controls would also reduce the likelihood of cross‐contamination from bulk packaging.

In the longer term, and based on the available risk information, a regulatory change may be pursued to provide improved authority for plant‐based feed ingredients. As with any proposed regulatory amendment, consultation with impacted industry stakeholders is a requirement.

This approach to risk management contained an appropriate degree of flexibility and supports the OIE principle that risk assessment be 'flexible to deal with the complexity of real life situations', including different 'types and amounts of data and information' (OIE, 2019). In the case of Canada, the import measures applied were seen to represent a measured approach to reduce an uncertain risk from comparatively higher risk commodities.

To summarize, the importation of relevant feed ingredients require that:

- The importer applies for an import permit in advance of the shipment
- Each import permit application be accompanied by a questionnaire completed by the Canadian processing facility or the processing facility in the country of origin, depending on the specific commodity
  ○ The questionnaire is necessary to demonstrate that the conditions of the import permit will be met (conditions such as adequate processing temperatures, hold times or procedures to minimize risk of cross‐contamination)
  ○ Example requirements from the questionnaires to be attested to include:
    ■ Verification of storage temperatures (where applicable)
    ■ Verification of treatment temperatures (where applicable)
    ■ Verification of process flows to reduce risk of cross‐contamination

A CFIA inspector may go on‐site at destination facilities to review records and to verify that the conditions of import have been met and that the information provided in the questionnaire is accurate.

For a complete overview of the import requirements for plant‐based feed, including access to the aforementioned questionnaires, please refer to: https://inspection.canada.ca/animal‐health/terrestrial‐animals/diseases/reportable/african‐swine‐fever/importers/eng/1556047524324/1556047524554

### 2.3 Risk communication

As per the OIE, risk communication 'is a multidimensional and iterative process and should ideally begin at the start of the risk analysis process and continue throughout' (OIE, 2019). This approach was exemplified as Canada worked through this challenging issue.

Participants in the pork and feed industries contributed to the analysis of risk throughout the process by providing information on industry practices and reviewing draft analyses. They also contributed to both risk assessment and risk management by participating in a collaborative working group. As part of the targeted compliance promotion and awareness activities, information was provided to importers of the highest risk ingredients in conjunction with outreach activities and a questionnaire administered in November 2018 and January 2019. All 31 importers of corn and soybeans were contacted, of which 12 indicated that they only import product of US origin. The remaining 19 answered questions on ASF awareness, source countries, suppliers, organic status, routine controls in place, packaging, secondary distribution, final end use, and feasibility of proposed controls. The results of the questionnaires indicated that industry supported the application of measures that were consistently applied, with no advantage or disadvantage being created. Consideration of this point resulted in improved industry acceptance as the process moved forwards.

Prior to initiation of the import requirements, a call was organized by industry to allow the CFIA to communicate the risk mitigation measures for select plant‐based feed ingredients with the industry sectors of other livestock species which may be potentially impacted by the changes. The implementation of new import requirements was documented in a notice to industry, issued 29 March 2019.

Communication occurred with the United States Department of Agriculture in relation to the development of the scenario tree, and the technical results of the analysis have been shared with risk assessors in Europe, the United States, and Australia.

Ongoing communication with industry and provincial stakeholders has occurred through regular teleconferences of the National Response Team for ASF.

## 3 CONCLUSION

The role of plant‐based feed in the transmission of ASF is not completely understood. However, even with an uncertain risk, countries such as Canada that would suffer severe consequences should ASF be introduced need to consider appropriate, risk‐based mitigation measures. The measures implemented should balance the effect of reducing the impact of an uncertain risk, with the costs and risks associated with the implementation. In this example, it is believed that Canada was successful in achieving this balance. It should also be emphasized that the early engagement of relevant stakeholders was a major contributing factor to the success of this initiative.

## CONFLICT OF INTEREST

The authors declare no conflict of interest.

## ETHICS STATEMENT

The authors confirm that the ethical policies of the journal, as noted on the journal's author guidelines page, have been adhered to. No ethical approval was required as this is a review article with no original research data.

## Data Availability

The data presented in this report are available on request from the corresponding author. The data are not publicly available due to privacy restrictions.
